# Multiobjective Optimization of Laser Polishing of Additively Manufactured Ti-6Al-4V Parts for Minimum Surface Roughness and Heat-Affected Zone

**DOI:** 10.3390/ma15093323

**Published:** 2022-05-05

**Authors:** Juliana S. Solheid, Ahmed Elkaseer, Torsten Wunsch, Steffen Scholz, Hans J. Seifert, Wilhelm Pfleging

**Affiliations:** 1 Institute for Applied Materials-Applied Materials Physics, Karlsruhe Institute of Technology, P.O. Box 3640, 76021 Karlsruhe, Germany; hans.seifert@kit.edu; 2 Institute for Automation and Applied Informatics, Karlsruhe Institute of Technology, 76344 Eggenstein-Leopoldshafen, Germany; ahmed.elkaseer@kit.edu (A.E.); steffen.scholz@kit.edu (S.S.); 3 Faculty of Engineering, Port Said University, Port Fuad 42526, Egypt; 4 Institute for Micro Process Engineering, Karlsruhe Institute of Technology, P.O. Box 3640, 76021 Karlsruhe, Germany; torsten.wunsch@kit.edu; 5 Karlsruhe Nano Micro Facility, Karlsruhe Institute of Technology, 76344 Eggenstein-Leopoldshafen, Germany

**Keywords:** laser polishing, Ti-6Al-4V, AM, surface quality, heat-affected zone, artificial neural networks, genetic algorithm, multiobjective optimization

## Abstract

Metal parts produced by additive manufacturing often require postprocessing to meet the specifications of the final product, which can make the process chain long and complex. Laser post-processes can be a valuable addition to conventional finishing methods. Laser polishing, specifically, is proving to be a great asset in improving the surface quality of parts in a relatively short time. For process development, experimental analysis can be extensive and expensive regarding the time requirement and laboratory facilities, while computational simulations demand the development of numerical models that, once validated, provide valuable tools for parameter optimization. In this work, experiments and simulations are performed based on the design of experiments to assess the effects of the parametric inputs on the resulting surface roughness and heat-affected zone depths. The data obtained are used to create both linear regression and artificial neural network models for each variable. The models with the best performance are then used in a multiobjective genetic algorithm optimization to establish combinations of parameters. The proposed approach successfully identifies an acceptable range of values for the given input parameters (laser power, focal offset, axial feed rate, number of repetitions, and scanning speed) to produce satisfactory values of Ra and HAZ simultaneously.

## 1. Introduction

Additive Manufacturing (AM) processes are widely applied in multiple sectors of industry because of their outstanding characteristics. Along with the design freedom it provides, its applicability to a broad variety of materials allows great flexibility in the production of simple or complex geometries with nearly no size restrictions. These attributes, in addition to shortened production lead times, make the method an attractive alternative to conventional manufacturing techniques for the aerospace, biomedical, and electronic industries. Every asset mentioned can be achieved with minimal influence on the cost and complexity of the manufacturing process [1,2]. This manufacturing process could play a major role in enabling a sustainable future for the AM industry in terms of resource consumption, waste management, and pollution control, reducing unwanted environmental impact, and it might, eventually, replace some conventional production methods [3].

Bearing in mind the extensive use of metallic materials in every industry, metal additive manufacturing (MAM) is currently one of the most relevant AM techniques. Among the fundamental approaches to building metallic parts, laser-powder bed fusion (LPBF) is the predominantly used MAM method because of its relative merits. However, despite the continued expansion of the variety of materials compatible with LPBF, the options are still constrained to castable and weldable metals and alloys [4]. The layer-by-layer nature of the fabrication process and the fixation of powder particles on the components’ surface can cause the formation of significant irregularities, the so-called dross formation [5]. Therefore, besides the improvement of mechanical behavior, major challenges for this manufacturing technique are the enhancement of surface quality and the refinement of the homogeneity of the microstructure. In order to achieve the required characteristics, LPBF parts are frequently subjected to postprocesses, such as heat treatment for microstructural alterations and shot peening, traditional machining, mechanical polishing, and sandblasting for surface quality [6,7,8,9,10]. Alternatively, nonconventional processes can be applied.

Considering the numerous systems and technologies available, along with the wide range of parameters that can be exploited for both AM and laser-polishing (LP) techniques, their different combinations provide significant versatility. During the laser-polishing process, the beam melts a thin superficial layer of the component. The consequent liquid metal flows evenly over the part because of surface tension, mitigating even major asperities. Therefore, the components’ roughness is diminished prior to the metals’ solidification. An exceptional characteristic inherent in this procedure is the lack of mechanical stresses, and this, together with its great efficiency and rapidity, makes it a feasible choice for finishing and enhancing the properties of AM parts. Moreover, LP is an adequate substitute for mechanical grinding and polishing [11,12,13,14]. However, the process might lead to deep heat-affected zones (HAZ), changing the material’s microstructure and mechanical properties because of the elevated temperatures involved [15,16]. For this reason, the HAZ depth must be carefully considered during the selection of relevant parameters. Furthermore, a better understanding of the fundamental mechanisms involved and changes in material properties will assist the decisions made regarding the process.

There are two distinct strategies that can be used to evaluate possible alterations in the component caused by laser polishing. The first relies on extensive experimentation with the need to prepare samples for analysis, whilst the second consists in developing a reliable model, substantially saving on material and time, especially when investigating a wide range of parameters. Amongst several options, the finite element method (FEM) is extensively used to provide a good understanding of process behavior. Using FEM, a numerical model capable of accurately describing the physical aspects of the procedure can be developed. Several researchers have explored the possibility of enhancing the performance of laser-polishing models in this way, varying the combination of heat transfer and fluid flow to predict surface properties, including the asperity of the initial surface and capillary flow [17,18,19]. Even though the method can require great computational time and complex models [20], it is an attractive strategy for the assessment of manufacturing processes. Moreover, the data obtained from the FEM can be combined with the data acquired from experimental analysis to develop optimization procedures [21].

From both experimental and simulated data, satisfactory process parameters can be identified via diverse established methodologies. The first possibility is to apply the popular “trial and error” approach, which is still extensively used even though it can require a great deal of time and expense. However, the success rate of the method generally relies on the personal experience of the machine operator. [22]. A common alternative is the combination of Design of Experiments (DoE) and statistical analysis to establish a regression model that describes the interaction between the parameters and how they influence the response. Compared with the “trial and error” methodology, this optimization procedure demands less experimentation/simulation to understand the effects of different parameters and determine predictions about process behavior. As a result of statistical analysis, an equation where the dependent variable (output) is stated as a function of the independent variables (input parameters) is acquired, allowing the estimation of the outcome for different values of each parameter. Even though the methodology can be employed in several fields, a particularly interesting application is its usage to optimize laser-based processes, such as laser cladding, welding, and surface hardening [23,24,25].

An additional option is to apply machine learning (ML) techniques to obtain similar models. The use of artificial neural networks (ANN), a fundamental ML method, for modeling the correlation between the input dataset and the desired outputs is increasing because of its great capability of adapting and learning from all kinds of data. Many studies in the literature have confirmed the efficacy of this approach for optimizing a broad variety of manufacturing processes [26,27,28]. 

As the name implies, ANN imitates the learning mechanism that takes place in the human brain, i.e., the way that the brain analyzes and processes information. Its architecture consists of input, hidden, and output layers that contain neurons linking the previous and following layers. Each neuron has a corresponding bias and each connection between them, also known as synapses, has an associated weight. Those biases and weights are updated throughout the training process, allowing adjustments until the model achieves a satisfactory fit to the data. Besides the proven effectiveness, a further benefit is the ability to receive new input data leading to an improvement in the model performance [29]. 

Having developed a mathematical model, the parameter optimization process can be finalized through standard methods. Since the aim of this work is to determine a range for each input parameter that will result in acceptable values for surface roughness and HAZ depth simultaneously, the elected approach must be able to solve multiobjective optimization problems, and the Genetic Algorithms (GA) was the chosen method. GA is one class of evolutionary algorithms and utilizes a metaheuristic procedure inspired by the natural selection concepts to find solutions for the optimization problem. Operators such as selection, crossover, and mutation ensure that the fittest individuals from a generic population will have their genomes recombined and mutated to create a new generation. This process is then repeated through the iterations until a satisfactory fitness level for the population is reached. Such characteristics make the GA a suitable approach to identify the Pareto front solutions for a multiobjective optimization problem, where the fittest individuals are determined not only by the fitness value (computed from the objective function) but also by the capacity to preserve or increase the diversity of the population in the Pareto frontier [30]. Even though the method does not guarantee a globally optimal solution, its stochastic nature permits the convergence to a large set of feasible solutions with less computational effort. Previously, the GA has produced excellent results when combined with ANN [31,32,33,34,35,36]. Furthermore, there is still a considerable research gap on approaches that not only combine GA multiobjective optimization and ANN models but also include data acquisition through both experiments and computational simulations for process development of the laser polishing.

In this context, further to some preliminary trials to identify the operating range and the effect of process parameters, the aim of the current research is to use experiments and simulations based on the same DoE to assess the effects of varying the laser-polishing parameters on the resulting surface roughness and HAZ depth. In particular, the approach presented here includes using the data obtained to create both linear regression and ANN models for each variable. The models with the best performance are then used in a multiobjective GA optimization to establish combinations of parameters that result in the simultaneous production of acceptable values of surface roughness and HAZ depth. The outcome of this paper is to assist in the understanding of how the parameters governing laser polishing can influence the final surface quality and how deeply it affects the material’s microstructure in doing so. This knowledge can lead to an informed decision on whether to use or not the laser-polishing process and at which step of the process chain to do so. Furthermore, it will offer an optimization tool for the selection of the appropriate laser-polishing process parameters, depending on specific predefined requirements of surface roughness and material properties. 

## 2. Materials and Methods

### 2.1. Sample Fabrication

**Figure 1:**
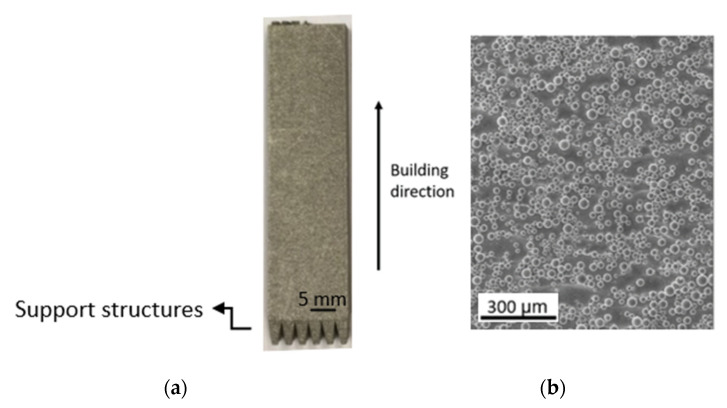
Ti-6Al-4V AM sample starting conditions: (**a**) photo of the block and (**b**) SEM image of the side surface.

Rectangular blocks of Ti-6Al-4V, with dimensions 55 × 15 × 4 mm^3^, were manufactured in the upright position via LPBF (Figure 1a) using a DMP Flex 350 with a maximum laser power of 500 W and wavelength of 1070 nm, operating in an inert, argon atmosphere. Scanning speed, laser power, and other parameters for the build process were set by the manufacturers. The resulting vertical surfaces’ (walls) high degree of roughness was due to powder attachment and dross formation during the layer-by-layer building process, see Figure 1b, with an initial surface roughness (Ra) of ~7 µm for all parts.

### 2.2. Laser System and Polishing Process

The laser polishing for surface quality improvement was executed ex situ using a Trumpf TruLaser Cell 3010 in combination with a TruDisk 3001 laser of wavelength 1064 nm operating in continuous mode with Argon injected across the face of the test piece to prevent oxidation. In this case, a lens with a focal length of 15 cm was used to focus the laser beam onto the sample surface. 

**Figure 2:**
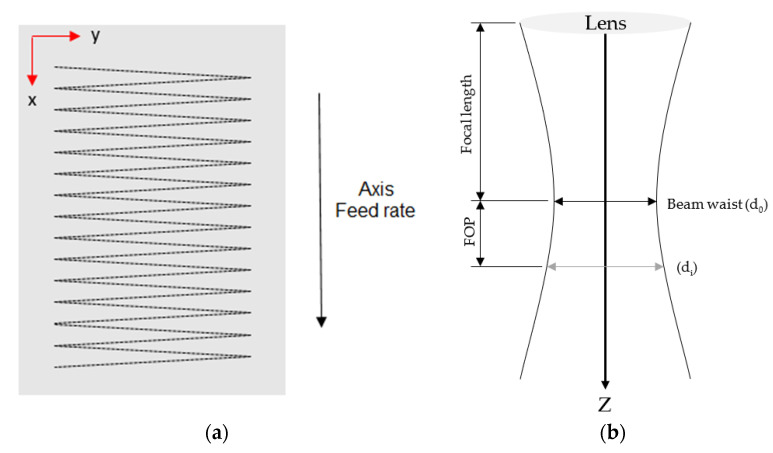
Schematic of: (**a**) laser scanning strategy and (**b**) laser spot size as a function of the focal offset.

To assess the performance of the laser-polishing process by this method, five parameters were selected: laser power, beam diameter, the number of repetitions (i.e., the total number of times the laser beam traverses the surface), axial feed rate, and scanning speed. Because of the pendulum-like movement of the scanning system (Figure 2a), the average scanning speed for the laser-polishing process with the given machine is not directly inputted. Instead, it is calculated based on the pendulum frequency and the adopted amplitude of displacement of the pendulum movement in the y-direction. Another governing parameter not directly inputted in the machine is the beam diameter, which is calculated based on the Rayleigh length and its dependence on the focal length. The focal offset (FOP), see Figure 2b, represents the distance between the laser focus and the surface to be processed. The diameter d_0_ is 0.1 mm (in focus, FOP = 0) when the disk laser is coupled with a 100 µm optical fiber [37]. Larger laser spot sizes are obtained with the variation of the focal position via focal offset (Figure 2b).

### 2.3. Surface Metrology System

The methods and procedures specified in EN ISO 4288 [38] were followed when measuring surface parameters, such as 2-D surface roughness, which necessitated stylus movement of 1.5 cm, evaluation length of 1.25 cm, length of sample 0.25 cm, and cutoff length of 0.25 cm. The instrumentation comprised a MarSurf XR 1 surface roughness measuring system, a MarSurf GD 26 driver, and a Kyocera BFW A 10-45-2/90 roughness probe. The measure selected as the characteristic to be used in subsequent assessments was Ra, the mean height of the surface asperities over the sampled length. 

### 2.4. Heat Transfer Model

Although laser polishing has the potential to reduce surface roughness, the heat it generates could alter the mechanical properties because of changes produced in the microstructure. The practical investigation of the effects of each set of parameters on the depth of the HAZ can be an extensive procedure, especially if the experiments are designed to generate large amounts of data. In this context, simulations of the laser-polishing process were performed on COMSOL Multiphysics 5.4 by means of a 3D heat transfer model, which has been presented and validated in a previous study [39].

**Figure 3:**
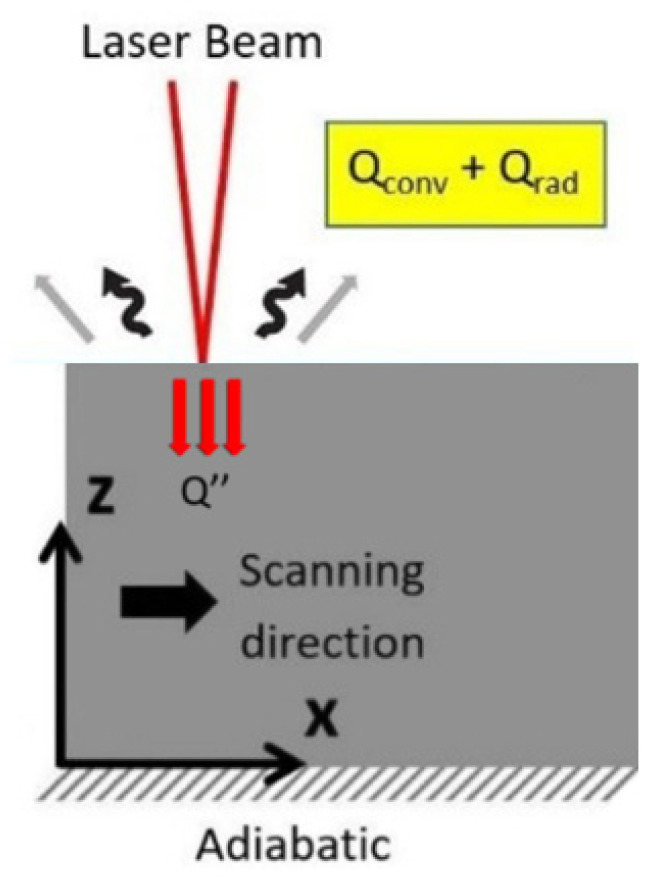
Schematic of the boundary conditions assumed in the model.

Figure 3 shows the thermal conditions assumed. The heat source, the scanning laser beam, is focused on the uppermost surface. Heat is lost from the surfaces of the material to the surrounding environment via radiation and convection. It is further assumed that no heat energy enters or leaves the bottom surface. 

Equation (1) is the equation governing heat transfer,

(1) $$\rho C_{p}\frac{\partial T}{\partial t}=k\nabla^{2}T$$

where k is the effective thermal conductivity, $C_{p}$ and ρ are the effective specific heat capacity and density of the material, respectively. 

Radiation and convection heat losses from the top surface and sides can be represented by Equations (2) and (3).

(2) $$Q_{rad}=\varepsilon \sigma \left(T_{amb}^{4}-T^{4}\right)$$

(3) $$Q_{conv}=h\left(T_{amb}-T\right)$$

where h the convective heat transfer coefficient, ε is surface emissivity, and σ is the Stefan–Boltzmann constant.

The generation of heat in the surface layer is represented by the laser acting as a point source with the heat flow having a Gaussian distribution,$\;Q^{\prime \prime }$, see Equation (4).

(4) $$Q^{\prime \prime }=\frac{2\alpha P_{w}}{\pi R_{w}^{2}}e^{\frac{-2\left(x^{2}+y^{2}\right)}{R_{w}^{2}}}$$

where $R_{w}$ is the distance from the center of the heat source, α is the laser absorption coefficient, and $P_{w}$ is the laser power.

Table 1 shows the values of thermophysical properties of Ti-6Al-4V and simulated process parameters.

The heat transfer will be greatly influenced by the fluid flow that occurs during melting, including the maximum temperatures attained [43]. However, to keep the time required for computation within reasonable limits, we did not consider the fluid flow directly. Instead, we adapted the thermal conductivity of the liquid in such a way that the melting process and depth of the HAZ corresponded to experimentally observed behavior. 

### 2.5. Design of Experiments

Initially, preliminary experimental trials were conducted to identify the working range of process parameters and their overall effect on the process responses in terms of generated surface topography and HAZ depth. However, by using DoE techniques, it is possible to define the individual and interactive effects of several factors that influence the experimental results. One advantage of adopting DoE is the possibility of identifying a set of experiments that will provide maximum information [44]. As stated above, five factors are considered the most important and were selected for the factorial central composite design to assess surface roughness, Ra; these were: laser power, beam diameter (focal offset), number of repetitions, axial feed rate, and scanning speed. Table 2 presents the ranges of the DoE parameter values used for the laser-polishing tests and simulations. Each factor had five levels, and from these, the DoE defined 52 experiments with 43 alternative parametric combinations and nine central repetitions.

The same design was adopted for modeling the depth of the HAZ resulting from the heat-transfer model described in the previous section. Since the model was developed for the simulation of laser tracks along only one axis, the parameter axial feed rate was excluded from the design. The final design resulted in 34 simulations and 25 different parameter combinations with 9 central repetitions. The factor levels were the same as for the laser-polishing experiments shown in Table 2.

#### 2.5.1. Linear Regression

The experimental Ra data and simulated HAZ data were processed with MATLAB (R2018b, MathWorks). A statistical model developed a quadratic expression to fit the data, see Equation (5).

(5) $$y=b_{0}+\sum b_{i}x_{i}+\sum b_{ii}x_{ii}^{2}+\sum b_{ij}x_{i}x_{j}$$

where ‘y’ corresponds to the objective function or output variable, ‘b_0_’,’b_i_’,’b_ii_’, and ‘b_ij_’ are the regression coefficients or predictors, ‘n’ is the number of factors, and ‘x_i_’ is the value of the ith factor. 

Least squares were used to estimate the regression coefficients, and a confidence level of 95% was chosen as the level of suitability of the proposed model.

#### 2.5.2. Artificial Neural Networks

**Figure 4:**
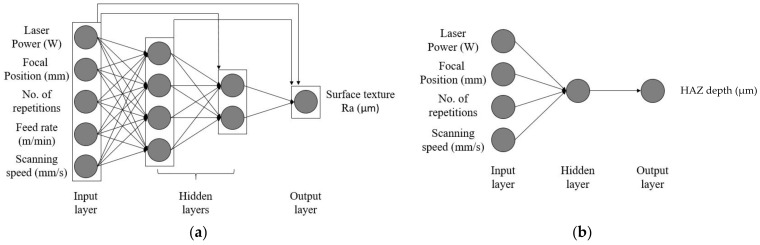
ANN architecture for (**a**) surface roughness and (**b**) HAZ depth.

Besides linear regression, this work also addresses the use of other approaches to achieve models with better performance for the same datasets. In this context, two individual architectures of ANN were employed to obtain models for estimating surface finish and HAZ using the Neural Network Toolbox in MATLAB. The optimal ANN was chosen using a least mean squares criterion from arrays of the hidden layers. A cascade-forward neural network composed of two hidden layers, containing 4 and 2 neurons each, respectively, was adopted for predicting the surface roughness of a given component. However, a much simpler feedforward structure gave satisfactory predictions for HAZ, with one hidden layer containing 1 neuron. The distinction between them is that the former connects all layers amongst them, from input to output, while the latter uses only regular synapses connecting one layer to the next. Figure 4 displays both architectures.

Both designs chosen used a sigmoid function (hyperbolic tangent) for the hidden layers and a linear transfer function for the output layers. A total of 70% of the datasets were selected on a random basis and then used as data for training; half the remainder was used for validation purposes, and the other half as data for testing. A damped least-squares backpropagation algorithm was chosen to train the ANNs. This approach interpolates between Gauss–Newton and gradient descent methods. When minimizing the sum of squared error function, the Hessian can be approximated, and this results in faster convergence with low values of mean square errors [45]. 

This is a robust method capable of learning from the input data, adjusting the model to new conditions, and concluding the procedure by predicting the outputs with significant accuracy. Such adaptation ability is achieved by weights associated with every synapse and a corresponding bias for each neuron. Those values keep changing throughout the training stage to obtain the most reliable model for the supplied sample set.

### 2.6. Optimization

Because laser polishing can result in both improvement of the LPBF surface quality and also undesired material modifications within the HAZ, the models obtained for surface roughness and depth of the HAZ are optimized simultaneously for minimum Ra and minimum HAZ via the built-in multiobjective GA in MATLAB. Besides favoring individuals with a better fitness value, such as in NSGA-II [46], a controlled elitist GA also favors individuals that preserve the diversity of the population, despite their fitness value [47]. Consequently, the optimization problem will converge to an optimal Pareto front, which will provide a set of noninferior solutions (i.e., it is possible to improve one objective only by degrading another). Thus, a suitable range for the parameters can be determined to achieve satisfactory values simultaneously for surface roughness and HAZ depth.

## 3. Results and Discussion

### 3.1. Initial Experimental Results

**Figure 5:**
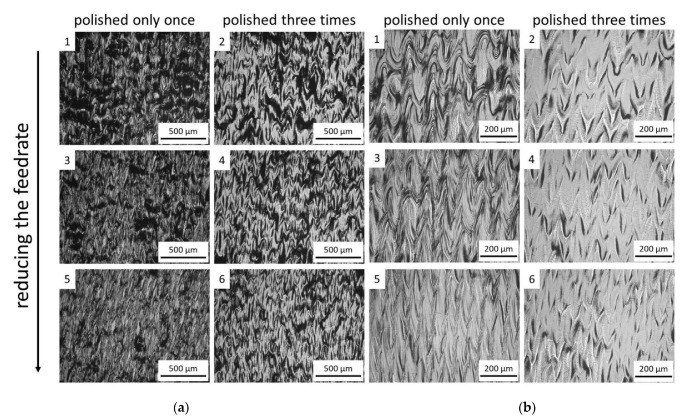
Optical microscopic images for the surface morphological analysis of samples processed with 100 W in two different magnifications (**a**) 20× and (**b**) 50×.

**Figure 6:**
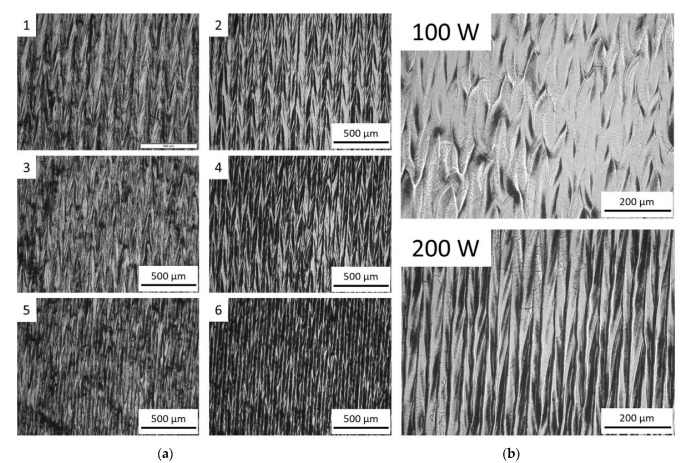
Optical microscopic images of (**a**) the surface morphological analysis of samples processed with 200 W and (**b**) comparison between samples treated with 100 W and 200 W.

**Figure 7:**
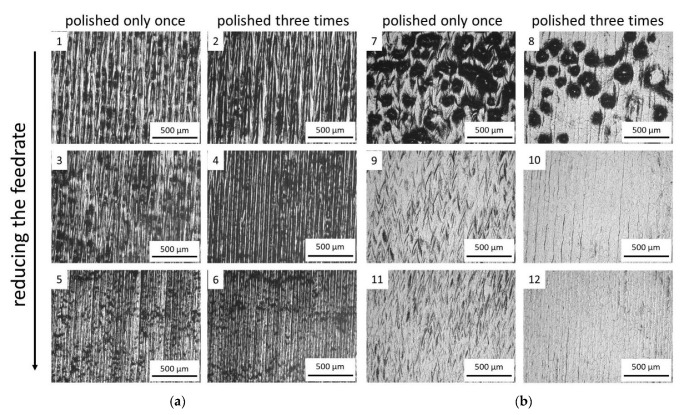
Optical microscopic images for the surface morphological analysis of samples processed with 300 W and (**a**) FOP = 0 mm and (**b**) FOP = +3 mm.

In this section, a detailed analysis of the surface topographies after laser polishing is conducted and qualitatively assessed for the identification of suitable process parameters working range. The topographies exhibited in Figure 5, Figure 6 and Figure 7 show that conducting the investigated process with higher laser power, lower axial feed rate, and with a higher number of repetitions could make a greater difference on the sample surface.

The higher energy input with the increasing power and number of repetitions caused more remelting of the material on the surface, which contributed to the smoothing of the sample surface. In contrast, the processed areas with lower energy input presented insufficient melting and no apparent improvement in the surface quality. The most evident cases of this observation are from samples exposed in Figure 5, in which the number of repetitions could not overcome the low energy input from the lowest laser power of 100 W. 

The resultant surface structure observed in Figure 5, after the remelting, presented a ripple form. The reason for the ripple formation is related to the rapid cooling rate of the laser polishing, in which the molten pools solidify rapidly after the irradiation of the laser spot. During the process, the laser spot continuously moves further to the unpolished areas and melts the new material. The previously melted material flows to the surrounding areas and solidifies when the energy input is no longer sufficient to maintain the liquid phase. In contrast, a small amount of material that absorbed most heat from the laser flows in a privileged direction because of the surface tension and inclination of the molten pool induced in the CW laser-polishing process. 

It is also observed that the degree of homogeneity was enhanced when the polishing process with the identical laser parameters was repeated three times in the same area. After triple repetitions, the number of ripple structures was reduced, which resulted in a more favorable surface condition. This improvement benefitted from the extra remelting process during the laser polishing in the second and third repetitions. In addition, indistinct scan tracks were noticed in Figure 5b. 

As shown in Figure 6a, the variations of the feed rate and repetition lead to the same impacts on the surfaces processed. When comparing the morphological analysis between the samples treated with 100 and 200 W (Figure 6b), with the only difference in the laser parameters between them being the laser power, the surface of the sample treated with higher power presented more homogeneous structures. The ripple form on the surface was elongated with the increased laser power from 100 W to 200 W. The borderlines of the scan tracks of the latter are more distinct than those on the former. It indicates that a more effective fusion occurred between the adjacent melt tracks when irradiated by the laser spot with a power of 200 W.

Finally, surfaces processed with 300 W were assessed (Figure 7). When the laser beam is in the focal offset of 0 mm, the ripple form on the surface of the sample could hardly be identified, and the surface tended to take shape in a groove form (Figure 7a). The distinct straight seam, related to the groove form, is observed between two adjacent scan tracks. The forming of this seam can be attributed to the hatch distance between the tracks during the laser scanning.

The effects of increasing the focal offset to +3 mm can be seen in Figure 7b. When comparing the two lowest feed rates, the adoption of a higher focal offset resulted in shallower grooves, although the straight seams are still evident. This occurs due to the reduced power density obtained with the defocus of the laser beam. With the highest feed rate, a particular surface feature was generated. Drop defects and craters are observed on the surfaces of samples 7 and 8. These defects originate from the insufficient melting of the surface material and other physical phenomena that occur during laser processing. Depending on the set of parameters, the lower power density can present beneficial results, such as the shallower groves, or result in catastrophic defects caused by insufficient heat input for a specific process.

### 3.2. DoE Experimental Results

**Figure 8:**
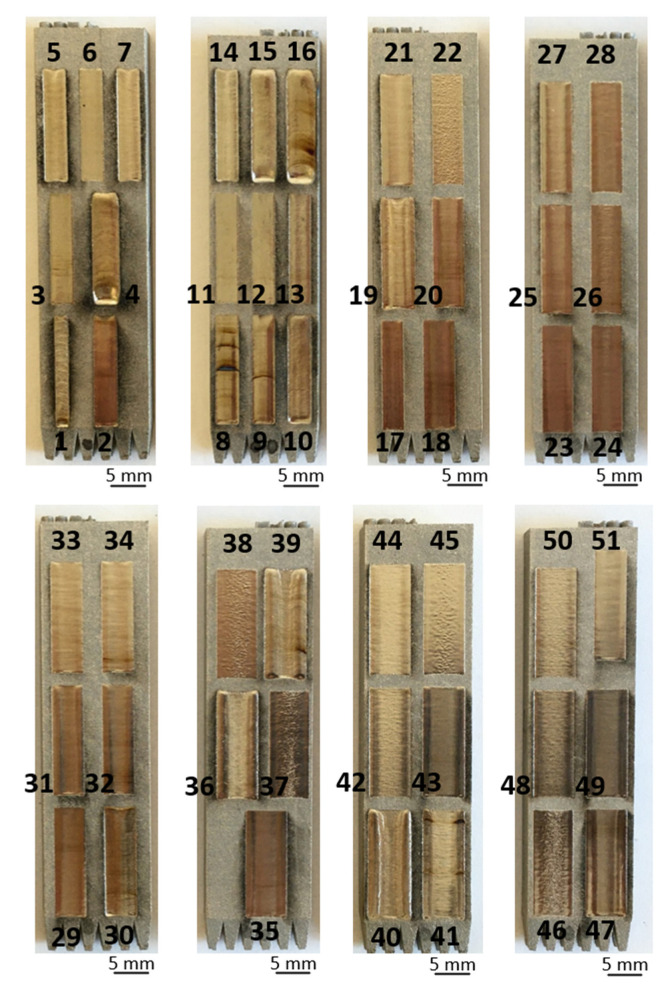
Overview image of DoE-based experimental results illustrating noticeable variations in the obtainable surface topographies based on the applied laser-polishing conditions.

In the present study, the surface roughness Ra data were acquired from experimental results. Overview images of the experiments can be observed in Figure 8. As seen in the pictures, varying the parameters in accordance with the DoE adopted led to a significant diversity of surface topographies with varied quality. Cases of overheating, insufficient remelting, and surface flattening can be easily noticed. Surface roughness quantitative results were obtained via the tactile measurement as formerly described in Section 2.3. The measured values for each of the 52 parameters variations with their correspondent standard deviation can be found in the supplementary material published with this work.

To vary the average scanning speed according to the experimental design adopted, the amplitude of displacement was changed accordingly, which resulted in discrepant processed areas in some cases. Because of the characteristics associated with the varied combination of parameters, such as material displacement and overheating, different sizes of the processed areas also occurred when the same amplitude of displacement was adopted.

**Figure 9:**
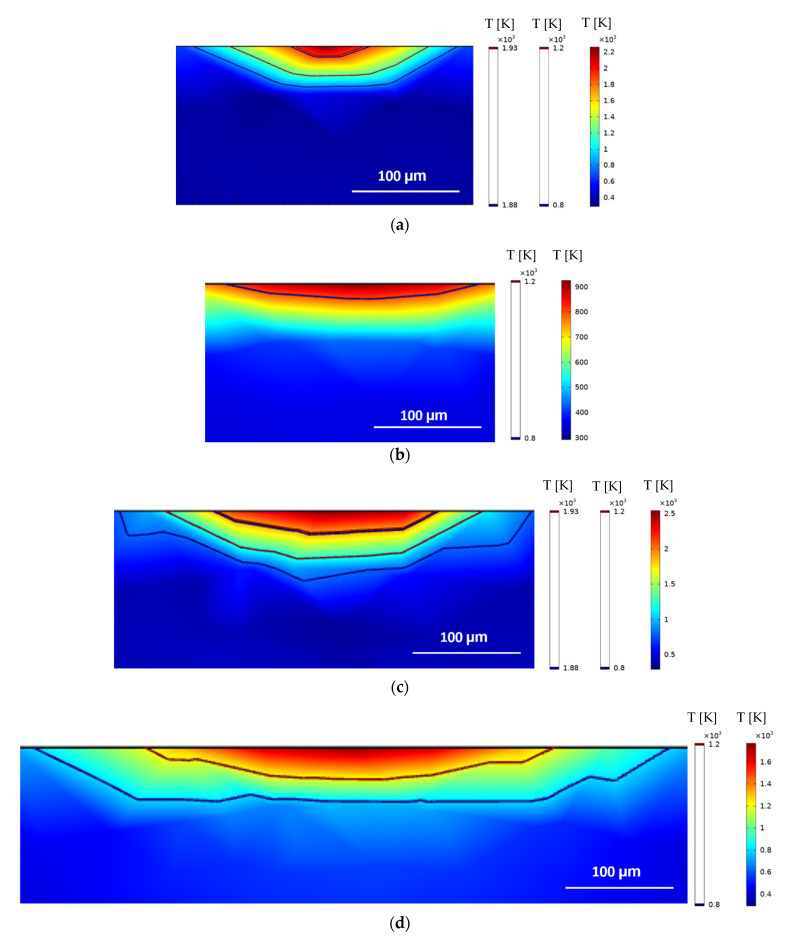
Simulated HAZ (1200 K contour) and melted (1930 K contour) areas of single-tracks laser polished with 1600 mm/s: (**a**) 200 W and +1 mm, (**b**) 200 W and +3 mm, (**c**) 400 W and +1 mm, (**d**) 400 W and +3 mm.

**Figure 10:**
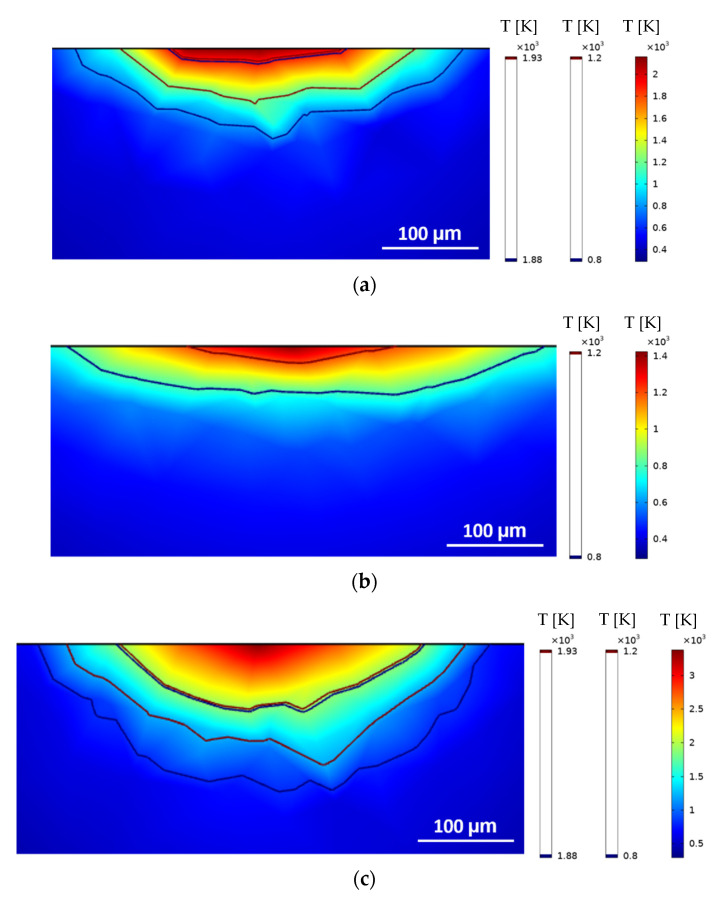

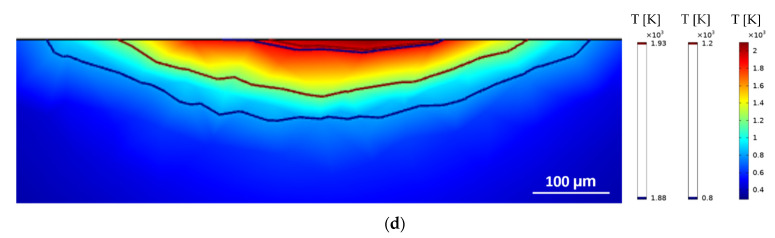
Simulated HAZ (1200 K contour) and melted (1930 K contour) areas of single-racks laser polished with 800 mm/s: (**a**) 200 W and +1 mm, (**b**) 200 W and +3 mm, (**c**) 400 W and +1 mm, (**d**) 400 W and +3 mm.

Following the validation of the implemented model via single-track experiments [39], the assessment of the HAZ depth for all 34 sets of parameters established in the DoE proposed in Section 2.4 is performed. Illustrations of some results obtained are presented in Figure 9 and Figure 10.

The heat transfer model adopted performs better for lower heat inputs because of the mesh size and distribution chosen to save computational efforts. This effect is observed in Figure 6 when the power is increased from 200 W to 400 W. Another evident aspect of using different sets of parameters that was accurately captured by the heat-transfer model is the laser–material interaction area and maximum temperatures achieved for different focal offsets. For 200 W with a +3 mm defocus, the maximum temperature achieved was slightly above 900 K and the contour delimitating the melted zone could not be identified, which implies that the intended remelting was not satisfactorily accomplished. For the same laser power and a lower defocus of +1 mm, the maximum temperatures achieved were above 2200 K, and the HAZ and melting contours are clearly identifiable. When maintaining the focal offset of +1 mm and increasing the power to 400 W, the HAZ contour presented was sharper but clearly deeper and broader, although the maximum temperature achieved was above 2500 K and did not represent a great increase. Finally, with 400 W and +3 mm, the effect of the defocusing on the laser beam diameter and the laser intensity distribution can be observed with the increased laser–material interaction area and reduced maximum temperature and HAZ depth.

As the scanning speed is reduced from 1600 mm/s to 800 mm/s, the parts are subjected to laser irradiation for a longer period; therefore, they are also subjected to higher heat inputs and present a higher maximum temperature. In this case, the sharp effects of the meshes are more common, as can be seen in Figure 10. Still, the overall depth of the HAZ can be estimated. The other discussed phenomena, such as laser–material interaction area, laser intensity distribution, and HAZ depth for different focal offset and laser power, present similar behaviors when comparing higher and reduced scanning speeds.

### 3.3. Linear Regression

This section addresses the development of the linear regression models for Ra and HAZ depth based on the experimental data obtained from the DoEs described above. It includes coefficient estimation, parameter interaction, and prediction capability of each model. 

#### 3.3.1. Model Coefficient Estimation

**Figure 11:**
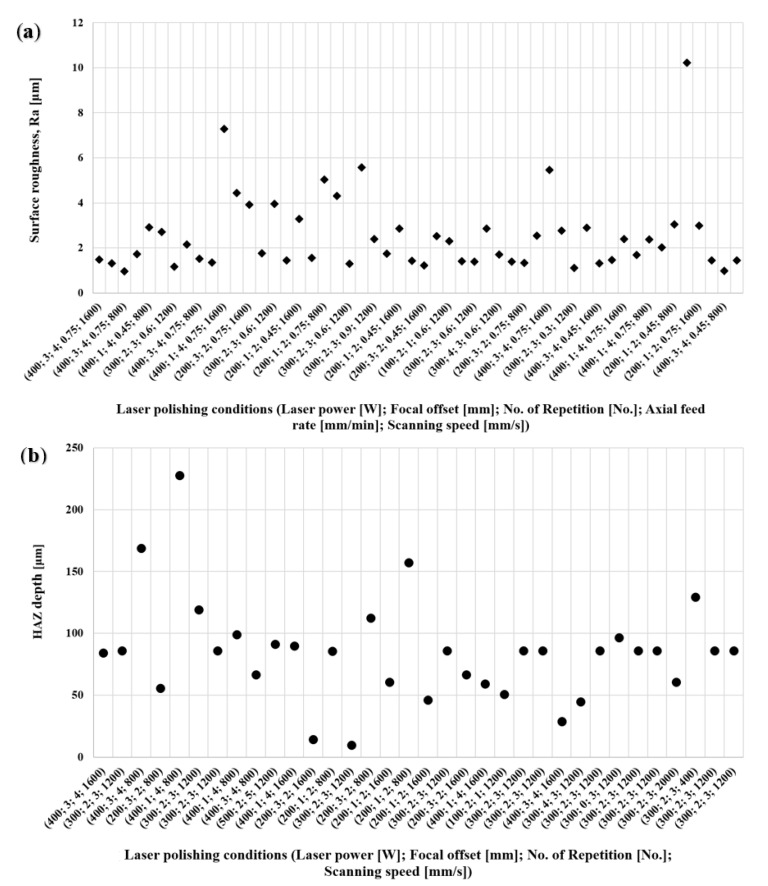
Obtained results for (**a**) surface roughness, Ra, and (**b**) HAZ depth.

A robust quadratic method was selected to obtain both regression models. Such a method has great resilience against outliers. Using this procedure, the coefficients in Equation (5) are established for Ra and HAZ depth. The data points acquired as presented in Section 3.1 and used as input into the algorithm are displayed in Figure 11.

In the first iteration, a similar weight is assigned to each data point, allowing the calculation of the coefficients via a conventional least-square approach. Subsequently, until the pre-established threshold is achieved, the subsequent iterations keep assigning new weights to each data point so that the values that are closer to the model’s prediction are favored with higher weight. Consequently, after convergence, the outcome is a model that is less sensitive to major alterations in the data than obtained via ordinary least squares.

The resulting models from the regression technique are in the form of quadratic equations. The first, Equation (6), provides a robust fit for surface roughness (µm), with initially 21 terms for the five chosen predictors. The final model presents 11 terms, which are the ones considered relevant.

(6) $$\begin{array}{ll} Ra= & 6.4603-0.014571x_{1}+0.4813x_{2}-1.2822x_{3}-6.0703x_{4}+0.028178x_{2}x_{3}\\ & -0.8623x_{2}x_{4}-0.38509x_{3}x_{4}+0.27358x_{2}^{2}+0.18903x_{3}^{2}+7.58x_{4}^{2} \end{array}$$

The second equation, Equation (7), is for the HAZ depth (µm) with initially 15 terms for only four predictors. The final model presents 10 terms, which are the ones considered relevant.

(7) $$\begin{array}{ll} HAZ\;depth= & 80.617+0.70384x_{1}-19.5x_{2}+7.9916x_{3}-0.15053x_{5}+0.021119x_{1}x_{2}\\ & +0.068898x_{1}x_{3}+2.908x_{2}x_{3}-3.6518x_{2}^{2}-3.5854x_{3}^{2} \end{array}$$

where ‘x_1_’ is the laser power, ‘x_2_’ is the focal offset, ‘x_3_’ is the number of repetitions, ‘x_4_’ is the axial feed rate, and ‘x_5_’ is the scanning speed.

From these equations, it is possible to obtain the interaction between different process parameters and predict Ra and the HAZ depths of components produced by AM and submitted for laser polishing using different sets of parameters. 

**Figure 12:**
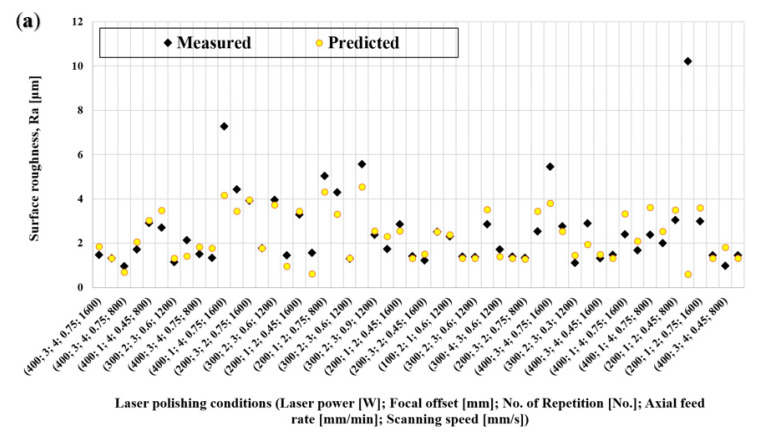

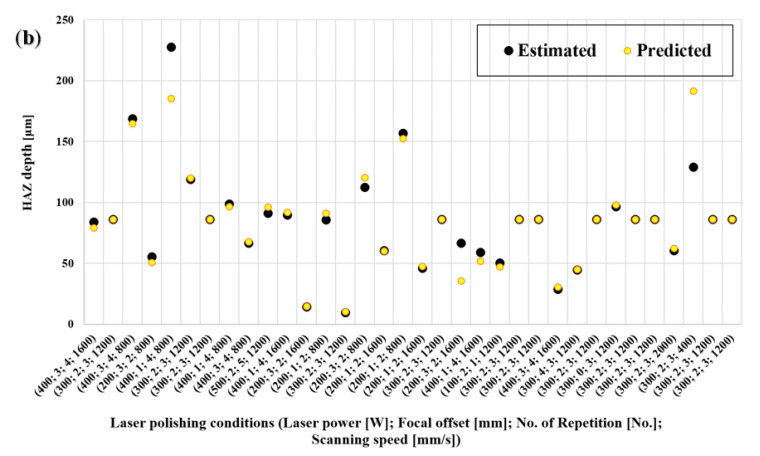
Predicted and measured values of (**a**) surface roughness regression and (**b**) HAZ depth regression.

The predicted values for each parameter set are shown in Figure 12, together with the measured values of Ra and depth of the HAZ.

The poor outcomes obtained for the Ra were associated with smaller data sets and the presence of high outliers; better results were obtained using the same procedure but with a larger set of input data [48]. The regression model for surface roughness has only the focal offset and the scanning speed statistically relevant at the 95% confidence level, the former being slightly more significant than the latter. The confidence levels for the axial feed rate, the number of repetitions, and the laser power were very low. However, the previously mentioned study resulted in a much better fit for the surface roughness model and showed the same order of parameter relevance as observed for the HAZ depth in the current work [48]. 

On the other hand, even with fewer input data points, a model giving a good fit was developed for the depth of the HAZ. Since the estimated heat-affected zone depths were obtained from a computational simulation without noisy data, the presence of outliers was not very likely amongst the input data sets. 

#### 3.3.2. Parameters Interaction

From the two models developed above, interaction plots were obtained. This was performed to provide a more detailed comparison between Ra and HAZ depth, respectively, with pairs of parameters. To maintain the commonality of the Ra and HAZ plots, the same pairs of parameters were used. The axial feed rate was excluded because it did not appear in the HAZ results and its contribution to Ra had a very low level of significance. 

**Figure 13:**
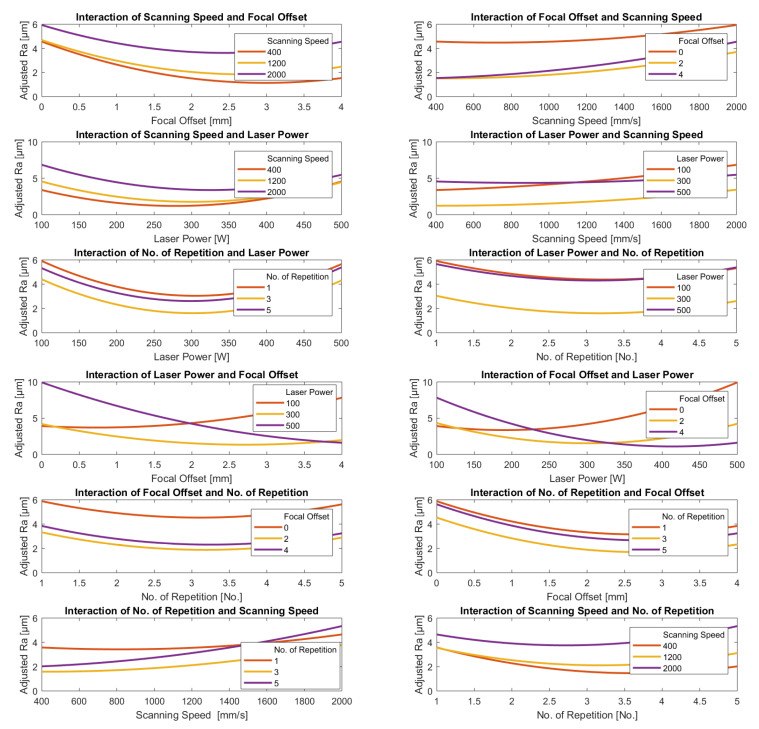
Interaction plots of the most significant laser-polishing parameters based on the surface roughness regression model.

**Figure 14:**
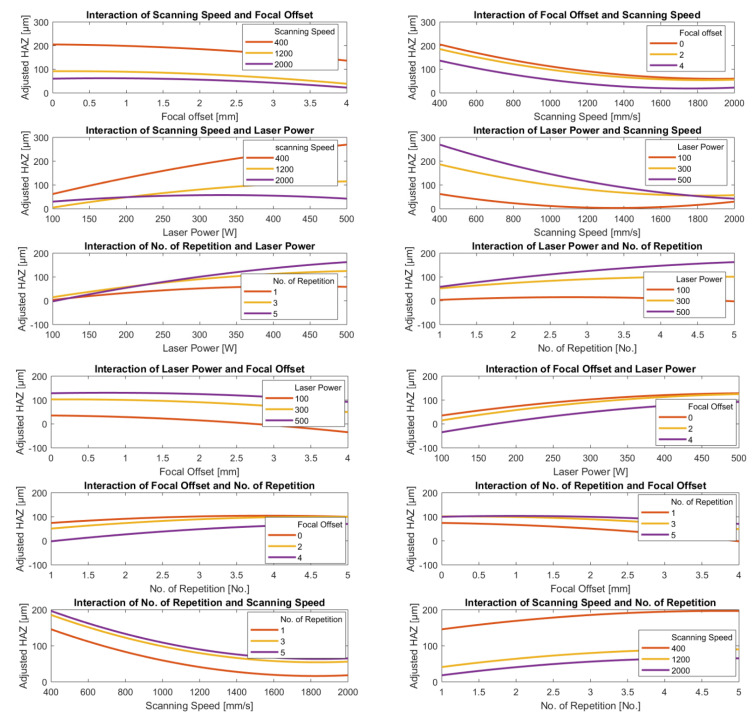
Interaction plots of the laser-polishing parameters for the HAZ depth regression model.

Figure 13 and Figure 14 show the variations in the adjusted Ra and HAZ depth, respectively, with pairs of parameters, each pair represented in two adjacent intersectional graphs. Each plot shows the predicted behavior of one parameter for each of the three levels of the other. In each plot, the remaining parameters are kept constant. The plots of the dependent variables plotted on the Y-axis in the following figures show how sensitive is its reaction to the independent variable plotted on the X-axis. With four separate parameters, there are twelve possible such combinations.

The interaction plots in Figure 13 show the clearest relation was between adjusted Ra and scanning speed; the lower the speed, the lower the value of the adjusted Ra for all values of power, the number of repetitions, and focal offset. There is also a clear tendency for the adjusted Ra to decrease as laser power increases with a broad minimum clearly visible around 300 W for both scanning speed and the number of repetitions, though the position of the minimum does tend to increase slightly with speed. However, minimum adjusted Ra is sensitive to change in focal offset. Minimum Ra occurs at higher values of the power the greater the value of the focal offset; for example, for the minimum value of the focal offset (0.0 mm), the minimum adjusted Ra occurs at a laser power of 100 W, but at the maximum value of the focal offset (+4.0 mm) the minimum adjusted Ra occurs at a laser power of 425 W. 

In all cases, the plot of the number of repetitions against any one of the other three parameters was a shallow “U” shaped curve with a minimum value of the adjusted Ra at the number of repetitions between 3 and 4.

The interaction plots in Figure 14 show that the estimated depth of the HAZ clearly decreases as laser power decreases and scanning speed increases for all values of focal offset and laser power and the number of repetitions, with the one exception that at the minimum power, 100 W, we observe a shallow “U” shaped curve with a minimum at about 1400 mm/s. The depth of the HAZ decreased slightly as focal offset increased but increased slightly as the number of repetitions increased, except in the case of higher power levels.

In contrast to the predicted behavior of the surface roughness, the general trends obtained from the HAZ depth model appear to be more generally applicable. The probable reason is the already mentioned data acquisition from the computational simulation without noise, which was adopted for HAZ depth estimation.

The above observations show the complexity of trying to identify the set of parameters that would deliver optimal values of Ra and HAZ depth simultaneously since most of the parameters for the two cases present conflicting consequences, i.e., it is only possible to reach the minimum Ra by degrading the HAZ depth, and vice versa. Therefore, any particular combination for low Ra and HAZ depth must be selected carefully, taking into account the specific circumstances.

In general, when using low laser power of 100 W or 200 W, the amount of energy supplied is insufficient to completely melt the surface asperities, possibly causing balling. Of course, the lower the laser power, the smaller the heat input to the surface and the less the depth of the HAZ. Reduced laser power can be compensated by increasing the number of repetitions or decreasing scanning speed and axial feed rate, which would increase the processing time and also the depth of the HAZ.

#### 3.3.3. Predictions

**Figure 15:**
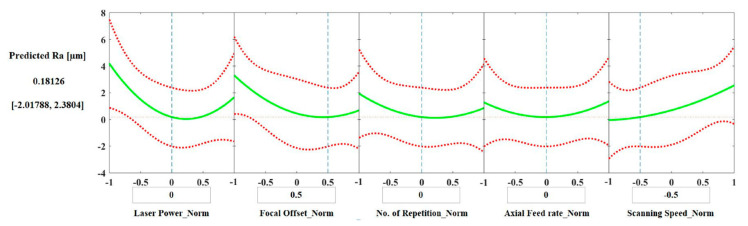
Surface roughness prediction slice plot with the normalized values for each input parameter.

**Figure 16:**
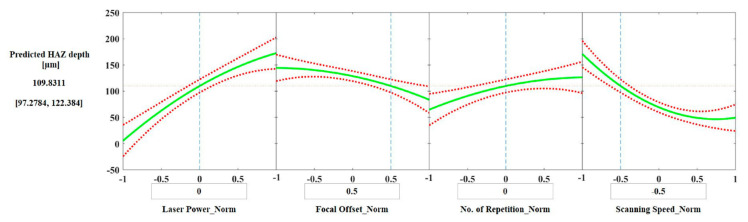
HAZ depth prediction slice plots with the normalized values for each input parameter.

From the regression analysis, it is possible to generate slice plots for predicting Ra and HAZ depth (Figure 15 and Figure 16), in which the curves represent how the dependent variable changes as a function of the values of the input parameters. The dashed boundaries are the 95% confidence limits for the predicted value of the dependent variable.

The slice plots allow the assessment of the predicted values of Ra and HAZ depth for a chosen set of parameters. For example, to obtain a laser polished surface with a Ra value of around 0.51 μm, the suggested set of parameters are: laser power = 0 (300 W), focal offset = 0.5 (+3 mm), number of repetitions = 0 (3 repetitions), axial feed rate = 0 (0.6 m/min), and scanning speed = −0.5 (800 mm/s). The same set of parameters would result in a HAZ depth of around 110 μm. 

Different applications may require a specific value of surface roughness but can accept a range of HAZ depths; thus, slice plots can be a useful tool to rapidly check the achievable varieties of surface roughness and HAZ depth in order to facilitate the design of further experiments since different combinations, besides the one presented above, will provide reductions in surface roughness and generate different depths of HAZ. 

Nevertheless, to predict several sets of parameters that will simultaneously provide satisfactory values of Ra and HAZ depth using only the slice plots can be time consuming and not always reliable, e.g., when the model is limited by the dataset and predicts unfeasible results (Figure 15). An alternative lies in building models based on machine learning techniques that can be improved with the addition of new data, whenever needed or available, and combined with numerical optimization approaches. The results would be more consistent models, and a wider range of optimal parameters obtained more quickly.

### 3.4. Artificial Neural Networks (ANN)

Since the regression model with the adopted DoE did not present satisfactory results for surface roughness prediction, this section will address the development and assessment of ANN architectures to model the relationship between input and output based on the experimental data. 

As mentioned in Section 2.5.2, the ANN design for surface roughness is composed of two hidden layers of four and two neurons, respectively, with a cascade forward network structure. A simple feedforward network architecture with one hidden layer of one neuron forms the ANN design for the HAZ depth. The Levenberg–Marquardt backpropagation algorithm, also described in Section 2.5.2, results in fast convergence with satisfactory values of MSE. The final models were obtained by learning from the input data, adjusting the model to new conditions using weights and bias associated with every synapse and each neuron. Those values were constantly updated during the training stage until the convergence threshold was accomplished.

**Figure 17:**
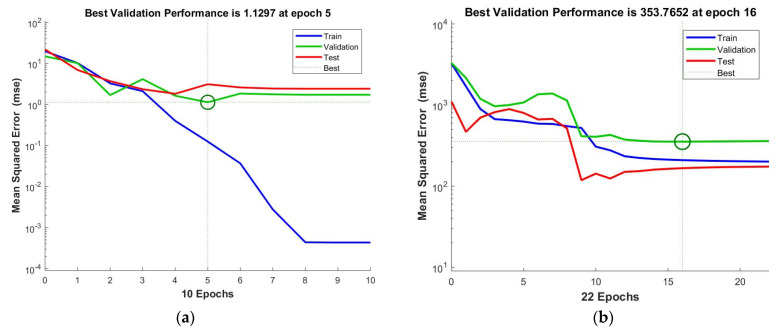
Validation performance of ANN models for (**a**) surface roughness and (**b**) HAZ depth.

The performance results for the training, test, and validation datasets for each model are presented in Figure 17. The performance is evaluated based on the MSE computed in every epoch. In most cases, during the training stage, the MSE has a tendency to decrease with the number of epochs executed. While the same is true for the validation stage, an increase in the MSE can occur if the network starts overfitting the training data. In order to avoid overfitting, the MATLAB ANN toolbox stops the training at a specified number of consecutive increases in the validation MSE; by default, this number is six. Overfitting can also be detected if the test MSE experiences a significant increase prior to the validation curve. As seen in Figure 17, for neither model, overfitting was an issue.

**Figure 18:**
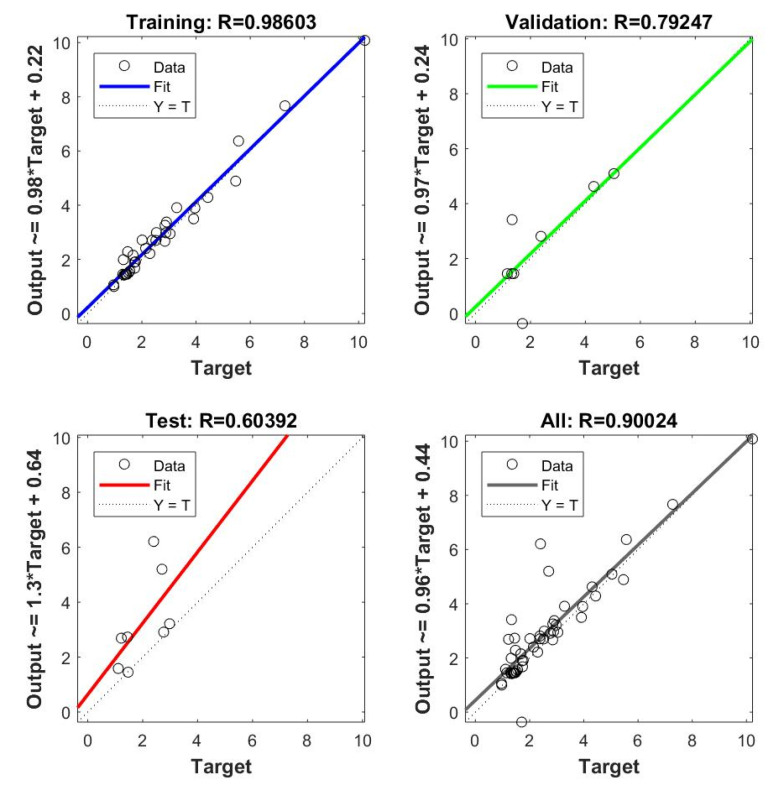
Regression plots from ANN model for surface roughness.

**Figure 19:**
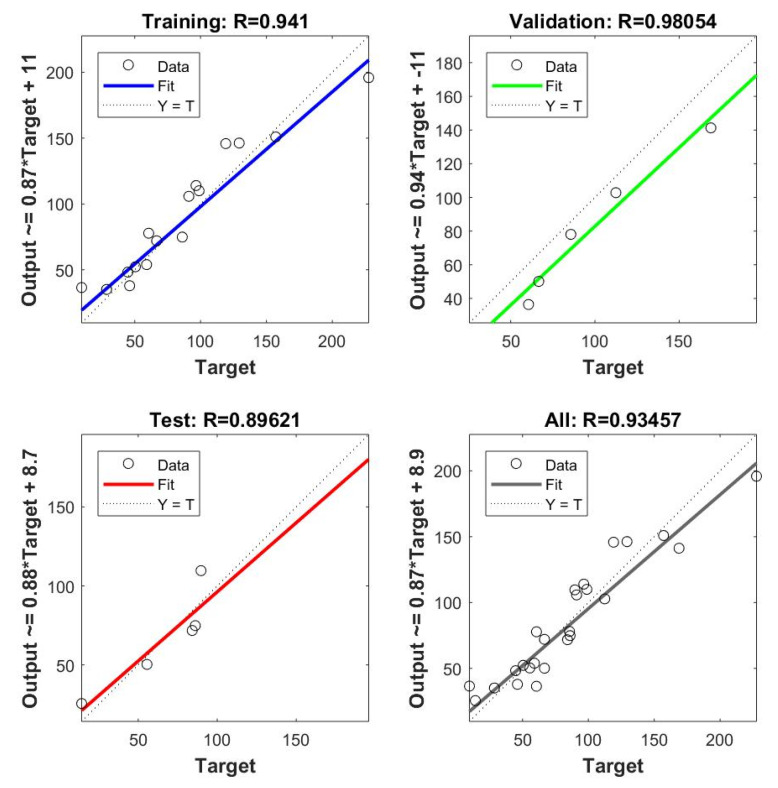
Regression plots from ANN model for HAZ depth.

From the regression plots (Figure 18 and Figure 19), the relationship between the predicted and measured output can be used to verify if the models are a suitable representation of the experimental data. Each image is divided into four, which are related to the training, validation, testing, and all data combined. Every subplot contains the distributed data points, a dashed line that demonstrates the perfect fit (R = 1) between output and targets, and a solid line that represents the actual fit. 

In the ANN model for surface roughness, the fit for the training data presented an R value of 0.98603, which indicates an almost perfect linear relationship. When considering the validation and test data, the R values obtained are 0.79247 and 0.60392, respectively. In addition to the different amounts of data used for each stage, 70%-15%-15% for training-validation-testing, the scatter plots lead us to believe that the validation and test datasets included a few outliers, unlike the training data set. However, the resulting fit for the entire dataset presents a satisfactory R of 0.90024. 

As for the ANN model for HAZ depth, the fit for the training data had an R value of 0.94100, while the validation and test data presented R values of 0.98054 and 0.89621, respectively. In this case, the absence of outliers resulted in a better fit for all stages despite the reduced amount of data, compared with the surface roughness. The overall R value for the HAZ depth ANN model for the entire dataset was R = 0.93457.

**Figure 20:**
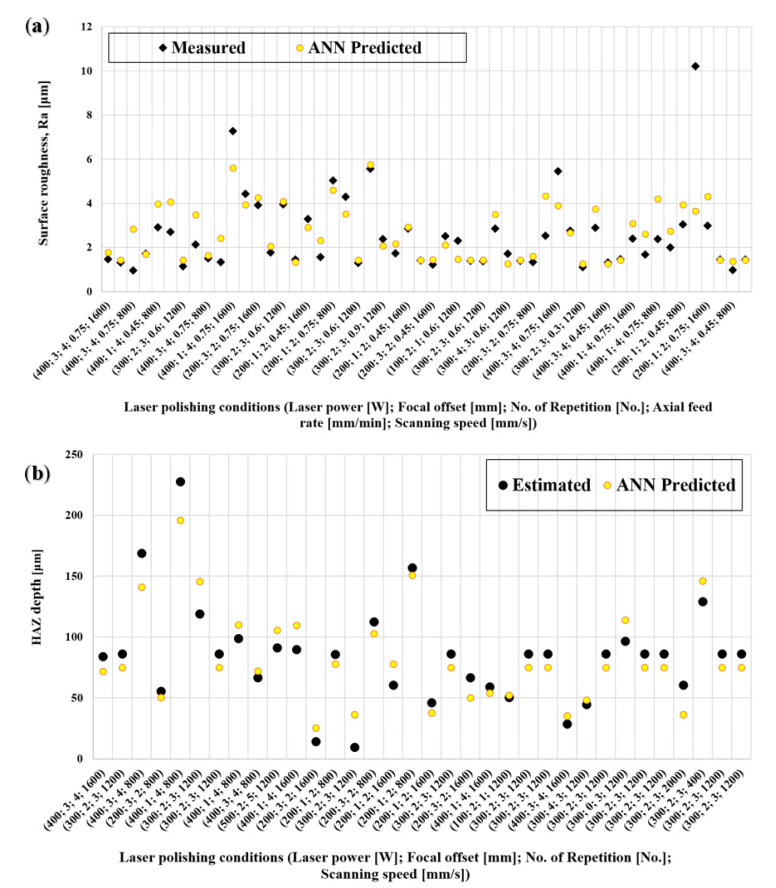
Comparison between ANN model predictions and measured values for (**a**) surface roughness and (**b**) HAZ depth.

To better illustrate the performance of the ANN model, the predicted values of Ra and HAZ depth are shown in Figure 20, together with the measured values. Table 3 presents the corresponding Mean Error, Mean Squared Error, and Standard Deviation associated with each model. In general, the ANN models display a better fit than the models obtained via quadratic regression. Considering the particular case for the Ra measurements, in contrast to the linear regression approach, the ANN technique was able to produce a satisfactory model for surface roughness prediction. For HAZ depth estimation, both methods provided an adequate match between predicted and measured values.

As can be seen from Figure 20, the model based on the ANN provides a good fit for the experimental input data, but it is also able to receive new data to improve its performance, which is not possible with regression analysis. For this reason, the ANN models were selected as the fitness function for the multiobjective optimization presented in the next section.

### 3.5. Multiobjective Optimization

**Figure 21:**
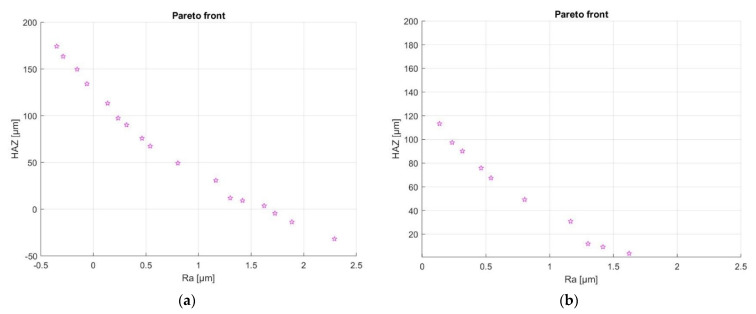
Pareto front containing (**a**) all optimal values and (**b**) realistic optimal values.

When facing a multiobjective problem, the goal is not to find a single solution but a set of acceptable solutions within a specific range. This set of solutions can be graphically represented in a Pareto front, where a set of points that have noninferior fitness function values is displayed in the parameter space. Figure 21a exhibits the Pareto front for the two competing objectives described in our work. However, not all solutions deliver realistic values for surface roughness and HAZ depth. The suitable solutions are displayed in Figure 21b, which is a zoom of the previous Pareto front restricted to the region of interest to this study.

The sets of parameters obtained to achieve the solutions displayed in the zoomed Pareto plot above (Figure 21b) are listed in Table 4. 

When performing multiobjective optimization, the values for one objective may improve while degrading the others. This is confirmed here. We see the minimum Ra occurs with the maximum HAZ depth and vice versa. The information obtained through GA optimization is also aligned with the parameter interaction presented in Section 3.3.2. For example, it was established in the interaction plots that to achieve minimum Ra and depth of HAZ, the focal offset should have the highest value of +4.0 mm. As is observed in Table 4 the focal offset is 3.99 mm (~4.0 mm) and is constant for all optimal solutions. On the other hand, parameters such as laser power, number of repetitions, and scanning speed present opposing behaviors to achieve minimum Ra and HAZ depth. In this aspect, the multiobjective optimization developed a balanced solution that consisted in combining high scanning speeds and a low number of repetitions, which tend to minimize the HAZ depth values, with laser power values closer to the optimal range to achieve minimum Ra. 

Considering the values of Ra and HAZ depth obtained, a suitable range for most applications would lie within or between solutions 2 to 9, 0.2 µm ≤ Ra ≤ 1.4 µm, with the corresponding range for the depth of HAZ being 10 µm ≤ HAZ ≤ 100 µm. 

We can conclude that acceptable ranges for each parameter would be: 

400 W ≤ laser power ≤ 460 W; 

Focal offset = +4.0 mm; 

Number of repetitions: 1, 2, or 3; 

0.58 m/min ≤ axial feed rate ≤ 0.68 m/min;

916 mm/s ≤ scanning speed ≤ 1380 mm/s.

## 4. Conclusions

The modeling approach presented here is suitable for multiobjective optimization of manufacturing processes, notwithstanding the particular variables to be optimized. The results presented here demonstrate that the adoption of DoE techniques can lead to the structured acquisition of data to be used in the development of statistical models. The first approach presented in this work is regression modeling, and it proved to be a valuable tool for assessing parametric interactions, despite the models developed having noticeable accuracy limitations. As an alternative and more flexible approach, ANN models were developed, and these resulted in simulations that were an acceptable fit for both surface roughness and depth of the HAZ. A particular strength of the ANN model is that it is capable of subsequently accepting additional data to assist in improving the model’s predictive capabilities.

Its slightly superior performance made the ANN model the better choice for fitness function in the GA multiobjective optimization, which provided an acceptable range of values for the given input parameters (laser power, focal offset, axial feed rate, number of repetitions, and scanning speed) to produce satisfactory values of Ra and HAZ, simultaneously. Given that the data used for training the ANNs were obtained from both experiments and numerical simulations, the proposed approach is a promising way to assist the optimization of additive manufacturing postprocesses, saving material, time, and money.

## Figures and Tables

**Table 1:** Thermophysical properties of Ti-6Al-4V and simulated process parameters, extracted from [40,41,42].

Property of Ti-6Al-4V	Value
Density (ρ)	4000 kg/m^3^
Solidus temperature (T_s_)	1878 K
Liquidus temperature (T_l_)	1928 K
Specific heat capacity, solid-phase (C_ps_)	543 J/kg K
Specific heat capacity, liquid-phase (C_pl_)	770 J/kg K
Thermal conductivity, solid-phase (k_s_)	13 W/m K
Thermal conductivity, liquid-phase (k_l_)	80 W/m K
Laser absorption coefficient (α)	0.3

**Table 2:** Ranges of values of DoE parameters used for the laser-polishing tests and simulations.

Laser Power (W)	Focal Offset (mm)	No. of Repetitions (No.)	Axial Feed Rate (m/min)	Scanning Speed (mm/s)
100	0	1	0.3	400
200	1	2	0.45	800
300	2	3	0.6	1200
400	3	4	0.75	1600
500	4	5	0.9	2000

**Table 3:** Mean Error, Mean Squared Error, and Standard Deviation associated with the ANN models adopted.

ANN Model	Mean Error	MSE	Std. Deviation
Ra	0.473481	0.737955	0.723771
HAZ	13.1259	224.302	7.32044

**Table 4:** Parameter sets to obtain adequate values of surface roughness and HAZ depth combined.

Solution	Ra (µm)	HAZ (µm)	Laser Power (W)	Focal Offset (mm)	No. of Repetitions	Axial Feed Rate (m/min)	Scanning Speed (mm/s)
1	0.13	113.31	410.16	3.99	2.52	0.65	795.14
2	0.23	97.35	410.84	3.99	2.59	0.64	916.60
3	0.31	90.15	413.47	3.98	2.66	0.66	993.97
4	0.46	75.82	407.15	3.99	2.36	0.66	1022.65
5	0.54	67.41	407.55	3.99	2.71	0.65	1180.17
6	0.80	49.22	436.31	3.99	2.61	0.64	1379.07
7	1.16	30.78	407.68	3.99	1.49	0.68	1159.97
8	1.30	11.92	459.39	3.98	1.47	0.58	1366.02
9	1.42	9.18	422.03	3.99	1.36	0.66	1343.78
10	1.62	3.68	437.22	3.99	1.08	0.68	1286.11

## Supplementary Materials

The following supporting information can be downloaded at: https://www.mdpi.com/article/10.3390/ma15093323/s1. The measured values for each of the 52 parameters variations with their correspondent standard deviation.
