# Supplementary material for: Multiobjective Optimization of Laser Polishing of Additively Manufactured Ti-6Al-4V Parts for Minimum Surface Roughness and Heat-Affected Zone
Source: Materials (Basel). 2022 May 5;15(9):3323. doi: 10.3390/ma15093323 (PMC9104100; doi:10.3390/ma15093323)
Supplement: Supplementary file 1 [file materials-15-03323-s001.zip › materials-1668669-supplementary.pdf]

Runs	Ra	Std dev
**1**	1,478	0,078
**2**	1,317	0,27
**3**	0,962	0,084
**4**	1,726	0,302
**5**	2,908	0,348
**6**	2,703	0,186
**7**	1,155	0,236
**8**	2,141	0,331
**9**	1,511	0,088
**10**	1,342	0,177
**11**	6,287	1,583
**12**	4,713	0,598
**13**	3,875	0,701
**14**	1,796	0,547
**15**	3,686	0,792
**16**	1,448	0,344
**17**	3,593	0,687
**18**	1,556	0,198
**19**	5,038	0,202
**20**	3,391	0,804
**21**	1,298	0,156
**22**	5,57	0,8
**23**	2,384	0,227
**24**	1,737	0,312
**25**	2,851	0,036
**26**	1,418	0,061
**27**	1,223	0,114
**28**	2,519	0,343
**29**	2,297	0,021
**30**	1,399	0,177
**31**	1,381	0,043
**32**	3,082	0,576
**33**	1,708	0,222
**34**	1,39	0,064
**35**	1,331	0,037
**36**	2,539	0,368
**37**	5,463	0,929
**38**	2,764	0,582
**39**	1,111	0,03
**40**	2,265	0,56
**41**	1,319	0,238
**42**	1,47	0,179
**43**	2,4	0,145
**44**	1,677	0,132
**45**	2,381	0,161
**46**	2,011	0,186
**47**	3,042	0,317
**48**	10,505	0,669

**49**	2,979	0,166
**50**	1,449	0,19
**51**	0,972	0,177
**52**	1,446	0,371
